# Improving the Accuracy of Two-Color Multiview (2CMV) Advanced Geospatial Information (AGI) Products Using Unsupervised Feature Learning and Optical Flow

**DOI:** 10.3390/s19112605

**Published:** 2019-06-08

**Authors:** Berkay Kanberoglu, David Frakes

**Affiliations:** 1 School of Electrical, Computer and Energy Engineering, Arizona State University, Tempe, AZ 85281, USA; 2 School of Biological and Health Systems Engineering, Arizona State University, Tempe, AZ 85281, USA; dfrakes@asu.edu

**Keywords:** SAR, 2CMV, change detection, optical flow, k-means, K-SVD

## Abstract

In two-color multiview (2CMV) advanced geospatial information (AGI) products, temporal changes in synthetic aperture radar (SAR) images acquired at different times are detected, colorized, and overlaid on an initial image such that new features are represented in cyan, and features that have disappeared are represented in red. Accurate detection of temporal changes in 2CMV AGI products can be challenging because of ’speckle noise’ susceptibility and false positives that result from small orientation differences between objects imaged at different times. Accordingly, 2CMV products are often dominated by colored pixels when changes are detected via simple pixel-wise cross-correlation. The state-of-the-art in SAR image processing demonstrates that generating efficient 2CMV products, while accounting for the aforementioned problem cases, has not been well addressed. We propose a methodology to address the aforementioned two problem cases. Before detecting temporal changes, speckle and smoothing filters mitigate the effects of speckle noise. To detect temporal changes, we propose using unsupervised feature learning algorithms in conjunction with optical flow algorithms that track the motion of objects across time in small regions of interest. The proposed framework for distinguishing between actual motion and misregistration can lead to more accurate and meaningful change detection and improve object extraction from an SAR AGI product.

## 1. Introduction

**Figure 1:**
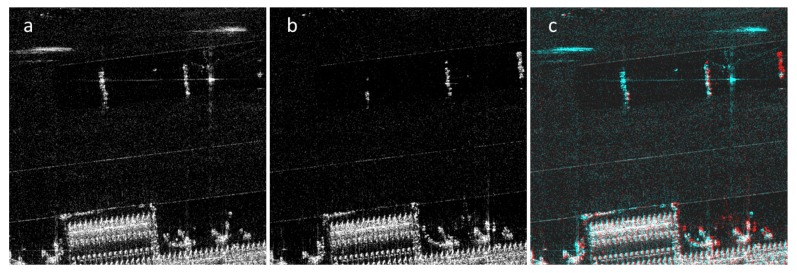
(**a**) reference image; (**b**) mission image; (**c**) two-color multiview (2CMV) image. In both images, there is an airplane visibly parked next to an airport building near the bottom center. In the second image (**b**), the airplane seems rotated by a small degree. The sharp edges of the building are slightly misregistered in the images and these registration errors are false positives in the 2CMV image.

One important use of synthetic aperture radar (SAR) imagery is in detecting changes between datasets from different imaging passes. Target and coherent change detection in SAR images have been extensively researched [1,2,3,4]. In two-color multiview (2CMV) advanced geospatial information (AGI) products, the changes are colorized and overlaid on an initial image such that new features are represented in cyan, and features that have disappeared are represented in red. In order to create the change maps, images are cross-correlated pixel-by-pixel to detect the changes. 2CMV products show changes at the pixel level and are often misleadingly dominated with red and cyan colors. Figure 1 shows a portion of a sample 2CMV image. In the sample images, there is an airplane visibly parked next to a building near the bottom center. It can be seen that many of the pixels in the 2CMV image are colored either red or cyan even if there is no change in the area.

Useful interpretation of temporal changes represented in 2CMV AGI products can be challenging because of speckle noise susceptibility and false positives that result from small orientation differences between objects imaged at different times. When every small intensity change creates a colored pixel, it becomes more difficult for operators and/or algorithms to detect meaningful changes and identify corresponding objects of interest.

In this work, we introduce a new framework of image processing methods for the efficient generation of 2CMV products toward extraction of advanced geospatial intelligence. Before false positive and object detection algorithms are performed, speckle and smoothing filters are used to mitigate the effects of speckle noise. Then, the number of false positive detections is reduced by applying: (1) unsupervised feature learning algorithms and (2) optical flow algorithms that track the motion of objects across time in small regions of interest.

There have been a number of change detection studies using thresholding [5,6,7,8], extreme learning machine [9,10], Markov random fields [11,12] and combinations of feature learning and clustering algorithms [13,14,15,16,17,18,19]. Optical flow fields can be used to distinguish between objects that have actually moved between frames and those that are in the same location but are slightly misregistered. Both cases of apparent motion can result in 2CMV detection, but they obviously differ greatly in terms of meaning. Investigation of the state-of-the-art in SAR image processing indicates that differentiating between these two general cases is a problem that has not been well addressed. Algorithms that mitigate speckle noise effects well and distinguishing between actual motion and misregistration can lead to better change detection. There is a lack of published methods for efficient generation of 2CMV products from SAR images, which serves as another motivating factor for this work.

The paper is organized in four sections. Following this introduction, Section 2 gives a brief background on the filtering, unsupervised feature learning, and optical flow techniques that were used and describes the stages of the proposed framework. Section 3 presents simulation results. Section 4 discusses the results and the contributions of the proposed methods.

## 2. Materials and Methods

In this section, we describe the key methods and steps of our image processing approach for generating change maps that drive the 2CMV representation and eliminating false positives in those maps.

### 2.1. Speckle Noise Filtering

Speckle noise is an inherent problem in SAR images [20] and causes difficulties for image interpretation by increasing the mean grey level of a local region. In order to mitigate speckle noise effects, we tested different speckle filter designs. Filters that were included in the testing were Frost [21], Enhanced Frost [22], Lee [23], Gamma-MAP [24], SRAD [25] and Non-Local Means [26]. In the end, Enhanced Frost filter was used in the algorithm due to its relatively straightforward implementation and comparable performance.

In [22], it was proposed to divide images into areas of three classes. The first class is comprised of homogeneous areas. The second class is comprised of heterogeneous areas wherein speckle noise is to be reduced, while preserving texture. The third class is comprised of areas containing isolated point targets that filtering should preserve. The Enhanced Frost filter output can be given as:

(1) $$\hat{I}\left(t_{o}\right)=\left\{\begin{array}{cc} \bar{I}, & \;\mathrm{for}\;C_{l}\left(t_{o}\right)<C_{u},\\ IK_{1}\exp\left[-K\left(C_{l}\left(t_{o}\right)-C_{u}\right)/\left(C_{max}-C_{l}\left(t_{o}\right)\right)|t|\right], & \;\mathrm{for}\;C_{u}\leq C_{l}\left(t_{o}\right)\leq C_{max},\\ I & \;\mathrm{for}\;C_{l}\left(t_{o}\right)\geq C_{max}, \end{array}\right.$$

where $t_{o}=\left(x_{o},y_{o}\right)$ is the spatial coordinate, $\bar{I}$ is the mean intensity value inside the kernel, *K* is the filter parameter, $K_{1}$ is a normalizing constant, and |t| is the absolute value of the pixel distance from the center of the kernel at $t_{o}$. The rest of the parameters are

$$\begin{array}{cc} C_{u} & =\sqrt{\frac{1}{L}},\\ C_{l}\left(t_{o}\right) & =\sigma /\bar{I},\;\mathrm{and}\\ C_{max} & =\sqrt{1+\frac{2}{L}}, \end{array}$$

where $C_{u}$ is the speckle coefficient of variation of the image, $C_{l}\left(t_{o}\right)$ is the local coefficient of variation of the filter kernel centered at $t_{o}$, $C_{max}$ is the upper speckle coefficient of variation of the image, and *L* is the number of looks. In our implementation, instead of *L*, we used “equivalent number of looks” (ENL). It can be defined as $ENL=\mu^{2}/\sigma^{2}$, where μ is the mean and σ is the standard deviation.

### 2.2. k-Means Clustering

The *k*-means clustering algorithm attempts to partition *p* observations into *k* clusters such that each observation belongs to the nearest cluster mean (centroid) [27]. The *k*-means algorithm iteratively tries to find *k* centroids for each cluster, while minimizing a within-cluster sum of squares

$$argmin\sum_{i=1}^{k}\sum_{x_{j}\epsilon S}{\left\|x_{j}-\mu_{j}\right\|}^{2},$$

where $x_{j}$ is the *j*^th^ observation and $\mu_{j}$ is the mean point (centroid) in the cluster. The basic steps of the algorithm are given in Algorithm 1:

**Algorithm 1***k*-means clustering algorithm
Initialize the centroids: Assign *k* points as the initial group centroids.Calculate the distance of each point to the centroids and assign the point to the cluster that has the closest centroid.After the assignment of all the points, recalculate the new values of the centroids.Repeat Steps 2 and 3 until the centroid locations converge to a fixed value.


### 2.3. K-SVD

K-SVD is a dictionary learning algorithm that is used for training overcomplete dictionaries for sparse representations of signals [28,29]. It is an iterative method that is a generalization of the *k*-means clustering algorithm. The K-SVD algorithm alternates between two stages: (1) sparse coding stage, and (2) dictionary update stage. In the first stage, a pursuit algorithm is used to sparsely code the input data based on the current dictionary. Based on Ref. [29], the Batch Orthogonal Matching Pursuit (Batch-OMP) algorithm can be used in this step. In the second stage, the dictionary atoms are updated to better fit the data via a singular value decomposition (SVD) approach. The basic steps of the K-SVD algorithm are given in Algorithm 2.

**Algorithm 2** K-SVD algorithm**Task:** Find the best dictionary to represent the data samples ${\left\{y_{i}\right\}}_{i=1}^{N}$, $y_{i}\epsilon R^{N}$ as sparse compositions by solving: $$min_{D,X}\left\{{\left\|Y-DX\right\|}_{F}^{2}\right\}\;\;\mathrm{subject}\;\mathrm{to}\;\forall_{i},{\left\|x_{i}\right\|}_{0}\leq T_{0}.$$
**Initialization:** Set the dictionary matrix $D^{\left(0\right)}$ϵ$R^{n\times K}$ with $l^{2}$ normalized columns. Set J=1.
**Iterations:** Repeat until convergence:Sparse coding stage: Use any pursuit algorithm to compute the representation vectors $x_{i}$ for each sample $y_{i}$ by approximating the solution of
$$i=1,2,\ldots ,N,\;min_{x_{i}}\left\{{\left\|y_{i}-Dx_{i}\right\|}_{2}^{2}\right\}\;\;\mathrm{subject}\;\mathrm{to}\;{\left\|x_{i}\right\|}_{0}\leq T_{0}.$$Dictionary update stage: For each column k=1,2,…,K in $D^{J-1}$,
-Define the group of samples that use this atom, $w_{k}=\left\{i\;|1\leq i\leq N,x_{T}^{k}\left(i\right)\neq 0\right\}$-Compute the overall representation error matrix, $E_{k}$, by
$$E_{k}=Y-\sum_{j\neq k}d_{j}x_{T}^{j}$$-Restrict $E_{k}$ by choosing only the columns corresponding to $w_{k}$, and obtain $E_{k}^{R}$.-Apply SVD decomposition $E_{k}^{R}=U\Delta V^{T}$. Choose the updated dictionary column $\tilde{d_{k}}$ to be the first column of *U*. Update the coefficient vector $x_{R}^{k}$ to be the first column of *V* multiplied by Δ(1,1).Set J=J+1.


### 2.4. Optical Flow

Optical flow is the apparent motion of objects in image sequences that results from relative motion between the objects and the imaging perspective. In one canonical optical flow paper [30], two kinds of constraints are introduced in order to estimate the optical flow: the smoothness constraint and the brightness constancy constraint. In this section, we give a brief overview of the optical flow algorithm we employ in the proposed methodology.

Optical flow methods estimate the motion between two consecutive image frames that were acquired at times *t* and *t* + δt. A flow vector for every pixel is calculated. The vectors represent approximations of image motion that are based in large part on local spatial derivatives. Since the flow velocity has two components, two constraints are needed to solve for it. The brightness constancy constraint assumes that the brightness of a small area in the image remains constant as the area moves from image to image. Image brightness at the point (*x,y*) in the image at time *t* is denoted here as I(x,y,t). If the point moves by δx and δy in time δt, then, according to the brightness constancy constraint:

(2) $$\frac{dI}{dt}=0.$$

This can also be stated as:

(3) I(r+δr,t+δt)=I(r,t),

where $r={\left(x,y,1\right)}^{T}$ and $r+\delta r={\left(x+\delta x,y+\delta y,1\right)}^{T}$. However, the brightness constancy constraint is restrictive. A less restrictive brightness constraint was chosen to address the intensity changes in SAR images. In Reference [31], it is proposed that the brightness constancy constraint can be replaced with a more general constraint that allows a linear transformation between the pixel brightness values. This way, the brightness change can be non-zero, or:

$$\frac{dI}{dt}\neq 0.$$

The formulation that allows a linear transformation between the pixel brightness values is less restrictive, and can be written as:

(4) I(r+δr,t+δt)=M(r,t)I(r,t)+C(r,t).

After using the Taylor series, the revised constraint equation can be obtained:

(5) $$I_{t}+I_{r}\cdot r_{t}-Im_{t}-c_{t}=0,$$

where $m_{t}=\lim_{\delta t\to 0}\frac{\delta m}{\delta t}$ and $c_{t}=\lim_{\delta t\to 0}\frac{\delta c}{\delta t}$.

The relaxed brightness constraint error is:

(6) $$\epsilon_{I}=\;\int \int \;{\left(I_{t}+I_{r}\cdot r_{t}-Im_{t}-c_{t}\right)}^{2}dx\;dy.$$

Equation (6) can be combined with the other constraint errors to produce the final functional to be minimized:

(7) $$\epsilon_{total}=\epsilon_{I}+\lambda_{s}\epsilon_{s}+\lambda_{m}\epsilon_{m}+\lambda_{c}\epsilon_{c},$$

where $\lambda_{s}$, $\lambda_{m}$, and $\lambda_{c}$ are error weighting coefficients. The remaining errors are given as:

$$\begin{array}{cc} \epsilon_{s} & =\;\int \int \;\|\nabla r_{t}{\|}_{2}^{2}dxdy,\\ \epsilon_{m} & =\;\int \int \;\|\nabla m_{t}{\|}_{2}^{2}dxdy,\\ \epsilon_{c} & =\;\int \int \;\|\nabla c_{t}{\|}_{2}^{2}dxdy. \end{array}$$

Substituting the approximated Laplacians into the Euler–Lagrange equations, a single matrix equation can be derived:

(8) $$\mathrm{Af}=g(\bar{f}),$$

where

$$A=\left(\begin{array}{cccc} I_{x}^{2}+\lambda_{s} & I_{x}I_{y} & -I_{x}I & -I_{x}\\ I_{x}I_{y} & I_{y}^{2}+\lambda_{s} & -I_{y}I & -I_{y}\\ -I_{x}I & -I_{y}I & I^{2}+\lambda_{m} & I\\ -I_{x} & -I_{y} & I & 1+\lambda_{c} \end{array}\right),f=\left(\begin{array}{c} u\\ v\\ m_{t}\\ c_{t} \end{array}\right),\;\;g\left(\bar{f}\right)=\left(\begin{array}{c} \lambda_{s}\bar{u}-I_{x}I_{t}\\ \lambda_{s}\bar{v}-I_{y}I_{t}\\ \lambda_{m}{\bar{m}}_{t}+I_{t}I\\ \lambda_{c}{\bar{c}}_{t}+I_{t} \end{array}\right).$$

These equations have to be solved iteratively. The solution is given by:

(9) $$f=A^{-1}g\left(\bar{f}\right),$$

where

$$A^{-1}=\frac{1}{\alpha }\left(\begin{array}{cccc} \begin{array}{c} \lambda_{c}\lambda_{m}\lambda_{s}+\lambda_{m}\lambda_{s}+\\ I^{2}\lambda_{c}\lambda_{s}+I_{y}^{2}\lambda_{c}\lambda_{m} \end{array} & -I_{x}I_{y}\lambda_{c}\lambda_{m} & I_{x}I\lambda_{c}\lambda_{s} & I_{x}\lambda_{m}\lambda_{s}\\ -I_{x}I_{y}\lambda_{c}\lambda_{m} & \begin{array}{c} \lambda_{c}\lambda_{m}\lambda_{s}+\lambda_{m}\lambda_{s}+\\ I^{2}\lambda_{c}\lambda_{s}+I_{y}^{2}\lambda_{c}\lambda_{m} \end{array} & I_{y}I\lambda_{c}\lambda_{s} & I_{y}\lambda_{m}\lambda_{s}\\ -I_{x}I\lambda_{c}\lambda_{s} & I_{y}I\lambda_{c}\lambda_{s} & \begin{array}{c} \left(I_{x}^{2}+I_{y}^{2}\right)\lambda_{c}\lambda_{s}+\\ \lambda_{c}\lambda_{s}^{2}+\lambda_{s}^{2} \end{array} & -I\lambda_{s}^{2}\\ I_{x}\lambda_{m}\lambda_{s} & I_{y}\lambda_{m}\lambda_{s} & -I\lambda_{s}^{2} & \begin{array}{c} \left(I_{x}^{2}+I_{y}^{2}\right)\lambda_{m}\lambda_{s}+\\ \lambda_{m}\lambda_{s}^{2}+I^{2}\lambda_{s}^{2} \end{array} \end{array}\right)$$

and

$$\alpha =\lambda_{m}\lambda_{s}^{2}+I^{2}\lambda_{c}\lambda_{s}^{2}+\left(I_{x}^{2}+I_{y}^{2}+\lambda_{s}\right)\lambda_{c}\lambda_{m}\lambda_{s}.$$

The equations can then be solved iteratively for other pixels with:

(10) $$f^{k+1}=A^{-1}g\left({\bar{f}}^{k}\right),$$

where *k* is the iteration number. This way the matrix $A^{-1}$ need only be computed once. More details about this optical flow algorithm can be found in Ref. [31].

### 2.5. Image Processing Steps

In this section, we describe the image processing approach for extracting change maps. The inputs are two registered SAR images of the same field of view that were taken at different times, i.e., “reference” image and “mission” image. Due to the large size of the images, images were divided into subimages for processing.

**Figure 2:**
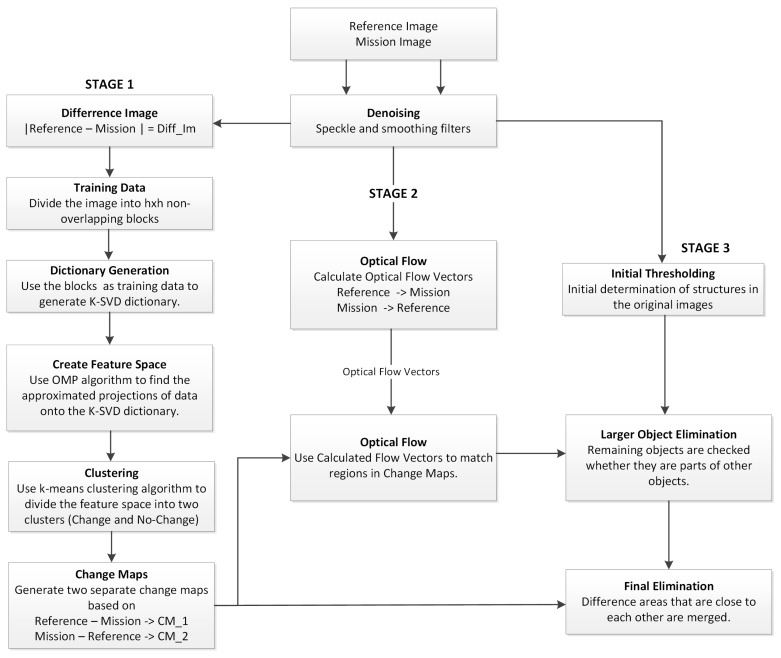
Flow diagram of the proposed framework.

In the denoising step, an Enhanced Frost filter, as described in Section 2.1, with a 5 × 5 window size was first used to mitigate the speckle noise effects. Then, a 9 × 9 low pass filter was used to smooth the test areas in order to obtain more uniform flow fields in the optical flow processing step. The remaining steps are grouped in three stages and described in the following subsections. The detailed flow diagram shown in Figure 2 can be used as a guide for the following descriptions.

#### 2.5.1. First Stage: Generation of Change Maps Using Unsupervised Feature Learning

**Figure 3:**
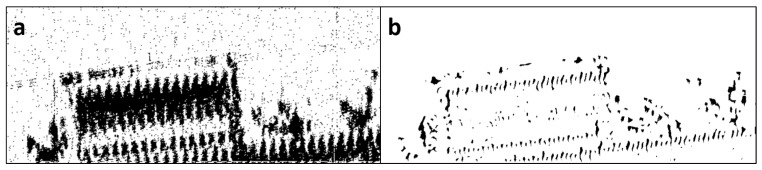
(**a**) change map with dictionary size = 30 atoms with 30 non-zero coefficients; (**b**) change map with dictionary size = 15 with three non-zero coefficients. Note that a larger dictionary size with more non-zero coefficients captures more changes.

Two change maps are needed for a 2CMV representation of an SAR image pair. Each change map represents the changes that exist in the corresponding SAR image. In this stage, we generate a combined change map and separate it into two change maps. In order to generate the combined change map, we used an approach similar to that was used in [13]. In the original approach, an eigenvector space is created by performing principle component analysis (PCA) on the difference image and k-means algorithm classifies the projections onto the eigenvector space into two classes: e.g., change and no-change. The basic steps are given in Algorithm 3. It should be noted that, in our framework, PCA was replaced with K-SVD because one can adjust the dictionary size and the sparsity constraint to obtain change maps with different levels of details. Figure 3 shows two change map results with different dictionary sizes.

**Algorithm 3** Generating change maps

**Difference Image:**
$$X_{dif}=\left|Reference-Mission\right|$$
**Training Data:** Divide $X_{dif}$ into hxh non-overlapping blocks.**Dictionary Generation:** Use the K-SVD algorithm to generate an overcomplete dictionary.
**Create Feature Space:**
-Generate hxh blocks for each pixel in $X_{dif}$ where the pixel is in the center of the block.-Use OMP algorithm to generate the projections of the data onto the dictionary.
**Clustering:** Use the *k*-means algorithm to classify the feature space into two classes, e.g. change and no-change.**Change maps:** Use the two classes to generate the combined change map. Divide the combined change map into two separate change maps based on the changes that occur in the images.


**Figure 4:**
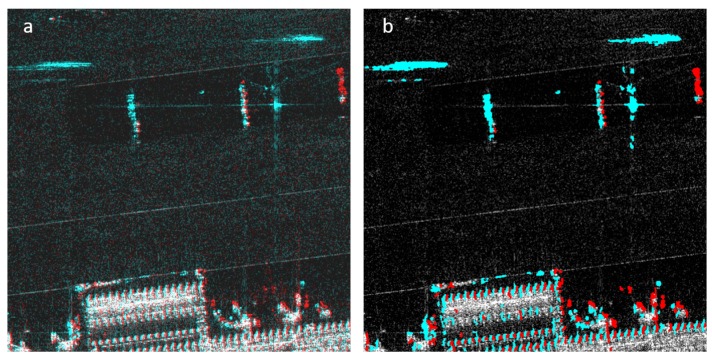
(**a**) original 2CMV image; (**b**) 2CMV image after Stage 1. Note that there are several false positives around the ridges of the building. In the second image, change colors (red and cyan) were made more pronounced to highlight the false positives.

After the change maps are generated, object properties such as area and location are calculated and, based on a user-defined area threshold, insignificant change areas are excluded from the change maps. The remaining change areas are then overlaid onto the reference image. In the 2CMV image, the areas that exist only in the reference image are colored in cyan and the areas that exist only in the mission image are colored in red. A sample 2CMV image after this stage is shown in Figure 4.

In a previous work, this stage was replaced by adaptive thresholding [32].

#### 2.5.2. Second Stage: Optical Flow

Figure 4 displays a 2CMV image after the first stage wherein it is clear that additional processing is needed to improve results because the ridges of the building in both images are slightly misregistered and they are shown as changes in both images. The primary improvement that is targeted with additional processing is reducing the number of false positives in the image. This goal can be accomplished with the use of the optical flow (OF) method described in Section 2.4. To manage computational complexity, the optical flow algorithm is performed on 256 × 256 pixel image blocks. Note that optical flow is calculated based on the original reference and mission images.

**Figure 5:**
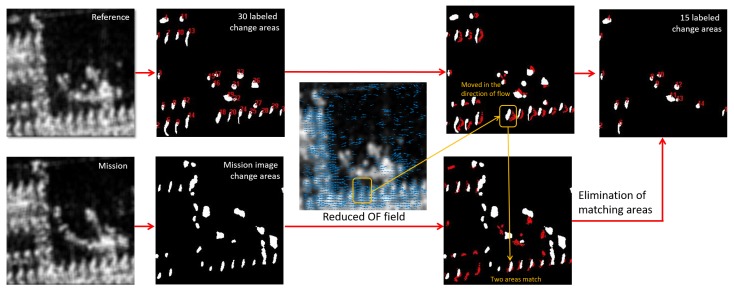
Elimination of false positives using optical flow. Change areas are moved along the flow direction in the reference image change map. Moved areas (shown in red) from the reference image are overlaid onto the mission image change map. The overlapped areas are then removed.

After obtaining the flow vectors, the direction of the majority of flow vectors is determined. The flow vectors that are in this direction are applied to the two first stage change maps to find matches. In the reference image, OF vectors are used to move the detected change areas in the flow direction. The destination of an area is then compared with the same location in the mission image. If there is a matching area based on location and size, then the two change areas are excluded from the change maps. The same process is performed in the opposite direction to match mission image change areas in the reference image. Figure 5 illustrates this step.

#### 2.5.3. Third Stage: OF Assisted Object Extraction

This stage has two main parts: extraction and elimination. Extraction is performed by an adaptive thresholding method that is similar to the one used in [32]. In this stage, the thresholding is performed on the original images to extract/label objects. The resulting two thresholded images are processed in two ways. First, OF vectors are used on the images to match the objects. The main difference from the second stage is that the flow vectors are used on the original thresholded images, not on the change maps. Change maps do not necessarily contain objects, and the goal is to find objects that moved between the two images. Objects with possibility of movement are labeled and compared against the areas in the change maps. It should also be noted that only some parts of an object can be detected as a change, and these detected changes can be used as a guide to extract the full object.

**Figure 6:**
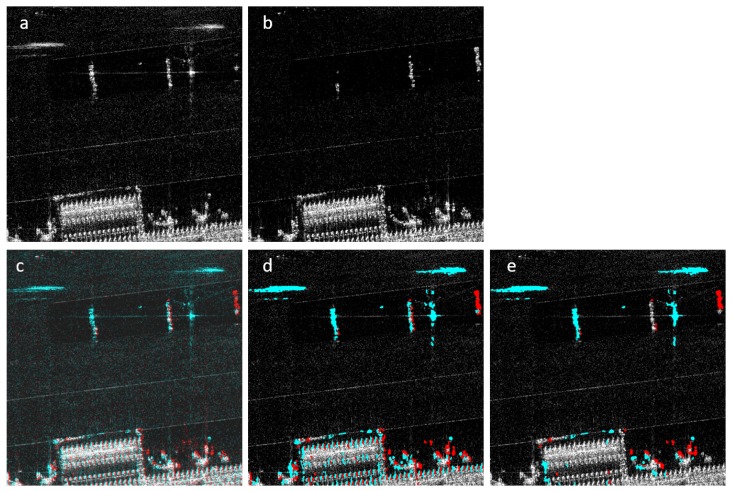
(**a**) reference image; (**b**) mission image; (**c**) original 2CMV image; (**d**) 2CMV image after using dictionary learning and clustering (Stage 1); (**e**) final 2CMV image. False positives are reduced.

After this process, the labeled areas in the change maps are overlaid on the reference image and checked whether they are a part of a larger object in the image. If the labeled area is found to be a part of a larger object, then the same location in the mission image is checked for the same object. In the case of two similar objects around the same location, it can be assumed that the detected object is a false negative and excluded from the difference map. After these two methods are performed, the output of this stage is generated by simply taking the intersection of the two results. Figure 6 shows how this process converts the reference image in (a) to the final output in (e).

## 3. Results

**Figure 7:**
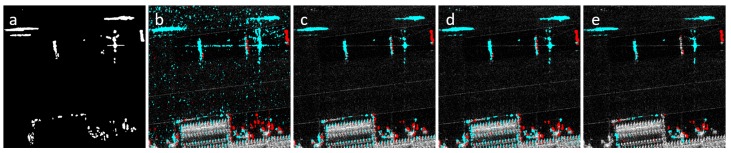
Results by (**a**) manual ground truth; (**b**) NR-ELM; (**c**) GaborTLC; (**d**) PCAKM; (**e**) proposed method.

The proposed algorithm was compared against three change detection methods: PCAKM [13], GaborTLC [18], and NR-ELM [10]. All three methods are implemented with their default parameters by using the publicly available code provided by the authors. The first dataset consisted of 1024 × 1024 regions from an SAR image pair provided by Lockheed Martin (Bethesda, MD, USA). The data were acquired with various Lockheed Martin SAR units, one example of which is an airborne long range, all weather, day/night, X-band SAR unit with a resolution of 1 m. The selected regions contained speckle noise and false positives that resulted from registration and perspective problems. 2CMV images were generated for each method. The visual results are shown in Figure 7. NR-ELM was more susceptible to noise compared to the other methods. It was noted that unsupervised dictionary learning and clustering algorithms were effective at removing false positives that did not match object profiles. Optical flow was effective for removing difficult false positives that resulted from registration and perspective problems.

From the ground truth map, the actual number of pixels belonging to the unchanged class and changed class are calculated, denoted as $N_{u}$ and $N_{c}$, respectively. With this information, five objective metrics are adopted for quantitative evaluation. False positive (FP) is the number of pixels belonging to the unchanged class but falsely classified as changed class. False negative (FN) is the number of pixels belonging to the changed class but falsely classified as unchanged class. The overall error (OE) is calculated by FP + FN. Percentage correct classification (PCC) and Kappa coefficient (KC) are as follows:

$$PCC=\frac{\left(N_{c}-FN\right)+\left(N_{u}-FP\right)}{N_{u}+N_{c}}\times 100\%,$$

$$KC=\frac{PCC-PRE}{1-PRE},$$

where proportional reduction in error (PRE) is defined as

$$\begin{array}{c} PRE=\frac{\left(N_{c}-FN+FP\right)\cdot N_{c}+\left(N_{u}-FP+FN\right)\cdot N_{u}}{{\left(N_{u}+N_{c}\right)}^{2}}. \end{array}$$

The results of the quantitative metrics are given in Table 1.

In addition to these results, the proposed framework was tested on an ensemble of 1024 × 1024 regions from the same SAR dataset. In many representative image regions where registration errors were prevalent, false positive detections were reduced by over 60%. Filtering of speckle noise and adaptive thresholds improved the quality of the object extraction and helped identify false positives. Establishing false positive motion/error thresholds, in accordance with initial image registration, can be key for continued improvement. It is also a challenge to extract only regions with intensity value changes. It is possible that wavelet based methods might be more successful with such a task.

**Figure 8:**
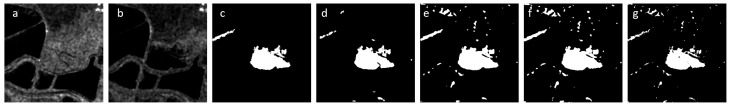
(**a**,**b**) San Francisco dataset; (**c**) ground truth; (**d**) NR-ELM; (**e**) GaborTLC; (**f**) PCAKM; (**g**) proposed method.

For the second test, a more standard dataset was used. The San Francisco dataset has been used in change detection studies and its ground truth change map was provided in [33]. It consists of two SAR images over the city of San Francisco that were acquired by ERS-2 C-band SAR sensor with VV polarization. The images were provided by the European Space Agency with a resolution of 25-m. These two images were captured in August 2003 and May 2004, respectively. The size of the images were 256 × 256 for this test. The change maps of the methods can be seen in Figure 8.

The results of the quantitative metrics are given in Table 2. The proposed framework performed comparable to PCAKM as a change detection algorithm. The San Francisco dataset doesn’t contain registration and perspective errors with speckle noise.

It should be noted that the proposed framework provided better results compared to the other methods when the datasets contain registration and perspective errors with speckle noise. Otherwise, the performance of the proposed method is comparable to PCAKM as a change detection algorithm since the optical flow processing stage cannot provide matching regions in the images.

Even though the computational complexity was not an issue during the course of this work, the speckle filtering, optical flow processing and merging are computationally expensive processes. On a dual core computer (Intel Core i7 6500U, Santa Clara, CA, USA) with 16 GB of memory, it takes slightly less than 3.5 min to process one region. There are many factors that are contributing to this time. Code was written in the MATLAB environment (R2016a, MathWorks, Natick, MA, USA) and not optimized for performance.

## 4. Conclusions

It was shown that unsupervised feature learning algorithms can be effectively used in conjunction with optical flow methods to generate 2CMV AGI products. Other image processing methods like noise reduction and adaptive thresholding were used to improve object extraction in the proposed methodology. Results demonstrated the ability of the techniques to reduce false positives by up to 60% in the provided SAR image pairs. However, there is still room for further improvement. For example, it was noticed that optical flow object matches close to image block borders can be overlooked due to the inaccuracy of flow vectors near the block borders. This problem can be addressed with a multigrid approach that leverages overlapping image blocks. Using this approach, if an object pair is close to the border in one block, then it will be near the center of an overlapping block. It has also been noted that only some parts of an object can be detected as a change, and the detected parts can be used as a guide to segment the full object. Objects that are close to one another can be merged to provide a more holistic analysis of the scene and further reduce the number of false positive object detections. However, it must be concurrently ensured that false positive reduction is not overly aggressive to the point that false negatives are generated. More recent optical flow or motion estimation algorithms can be investigated as an alternative to the one utilized in this work. The chosen optical flow method is suitable for the tested dataset and performs adequately as expected since it takes into account the intensity changes between images. The choice of K-SVD over PCA increased the computational complexity while allowing flexibility over the details of the change maps by changing the dictionary size and the number of non-zero coefficients. Dictionaries with higher number of non-zero coefficients provided more detailed change maps. For future work, investigating the correlation between the quantitative metrics and the parameters in the framework (e.g., dictionary size, etc.) can provide insight into tuning the framework for different types of datasets. Other methods can be researched as alternatives to the K-SVD method in the framework.

## Figures and Tables

**Table 1:** Results for the SAR dataset.

Methods	FP	FN	OE	PCC (%)	KC (%)	Time (s)
NR-ELM	97377	4276	101653	0.9031	0.2993	202.1
GaborTLC	20160	8449	28609	0.9727	0.5809	74.6
PCAKM	35135	6251	41386	0.9605	0.51	6.4
Proposed method	3865	12852	16717	0.9841	0.6569	199.1

**Table 2:** Results for the San Francisco dataset.

Methods	FP	FN	OE	PCC (%)	KC (%)
NR-ELM	328	440	768	0.9883	0.9107
GaborTLC	1376	60	1436	0.9781	0.8539
PCAKM	1855	73	1928	0.9706	0.8115
Proposed method	836	685	1521	0.9768	0.8277

## References

[1] El-Darymli K., McGuire P., Power D., Moloney C. (2013). Target Detection in Synthetic Aperture Radar Imagery: A State-of-the-Art Survey. J. Appl. Remote Sens..

[2] El-Darymli K., Gill E.W., McGuire P., Power D., Moloney C. (2016). Automatic Target Detection in Synthetic Aperture Radar Imagery: A State-of-the-Art Review. IEEE Access.

[3] Ashok H.G., Patil D.R. Survey on Change Detection in SAR Images. Proceedings of the IJCA Proceedings on National Conference on Emerging Trends in Computer Technology.

[4] Ren W., Song J., Tian S., Wu W. Survey on Unsupervised Change Detection Techniques in SAR Images1. Proceedings of the 2014 IEEE China Summit International Conference on Signal and Information Processing (ChinaSIP).

[5] Bazi Y., Bruzzone L., Melgani F. (2005). An Unsupervised Approach Based on the Generalized Gaussian Model to Automatic Change Detection in Multitemporal SAR Images. IEEE Trans. Geosci. Remote Sens..

[6] Bovolo F., Bruzzone L. (2005). A Detail-Preserving Scale-Driven Approach to Change Detection in Multitemporal SAR Images. IEEE Trans. Geosci. Remote Sens..

[7] Moser G., Serpico S.B. (2006). Generalized Minimum-Error Thresholding for Unsupervised Change Detection from SAR Amplitude Imagery. IEEE Trans. Geosci. Remote Sens..

[8] Sumaiya M.N., Kumari R.S.S. (2016). Logarithmic Mean-Based Thresholding for SAR Image Change Detection. IEEE Geosci. Remote Sens. Lett..

[9] Jia L., Li M., Zhang P., Wu Y. (2016). SAR Image Change Detection Based on Correlation Kernel and Multistage Extreme Learning Machine. IEEE Trans. Geosci. Remote Sens..

[10] Gao F., Dong J., Li B., Xu Q., Xie C. (2016). Change Detection from Synthetic Aperture Radar Images Based on Neighborhood-Based Ratio and Extreme Learning Machine. J. Appl. Remote Sens..

[11] Melgani F., Bazi Y. (2006). Markovian Fusion Approach to Robust Unsupervised Change Detection in Remotely Sensed Imagery. IEEE Geosci. Remote Sens. Lett..

[12] Yousif O., Ban Y. (2014). Improving SAR-Based Urban Change Detection by Combining MAP-MRF Classifier and Nonlocal Means Similarity Weights. IEEE J. Sel. Top. Appl. Earth Observ..

[13] Celik T. (2009). Unsupervised Change Detection in Satellite Images Using Principal Component Analysis and k-Means Clustering. IEEE Geosci. Remote Sens. Lett..

[14] Li W., Chen J., Yang P., Sun H. Multitemporal SAR Images Change Detection Based on Joint Sparse Representation of Pair Dictionaries. Proceedings of the 2012 IEEE International Geoscience and Remote Sensing Symposium.

[15] Lu X., Yuan Y., Zheng X. (2017). Joint Dictionary Learning for Multispectral Change Detection. IEEE Trans. Cybern..

[16] Ghosh A., Mishra N., Ghosh S. (2011). Fuzzy Clustering Algorithms for Unsupervised Change Detection in Remote Sensing Images. Inf. Sci..

[17] Nguyen L.H., Tran T.D. A Sparsity-Driven Joint Image Registration and Change Detection Technique for SAR Imagery. Proceedings of the 2010 IEEE International Conference on Acoustics, Speech and Signal Processing.

[18] Li H., Celik T., Longbotham N., Emery W.J. (2015). Gabor Feature Based Unsupervised Change Detection of Multitemporal SAR Images Based on Two-Level Clustering. IEEE Geosci. Remote Sens. Lett..

[19] Gong M., Su L., Jia M., Chen W. (2014). Fuzzy Clustering With a Modified MRF Energy Function for Change Detection in Synthetic Aperture Radar Images. IEEE Trans. Fuzzy Syst..

[20] Dekker R.J. (1998). Speckle Filtering in Satellite SAR Change Detection Imagery. Int. J. Remote Sens..

[21] Frost V., Stiles J.A., Shanmugan K.S., Holtzman J.C. (1982). A Model for Radar Images and Its Application to Adaptive Digital Filtering of Multiplicative Noise. IEEE Trans. Pattern Anal. Mach. Intell..

[22] Lopes A., Touzi R., Nezry E. (1990). Adaptive Speckle Filters and Scene Heterogeneity. IEEE Trans. Geosci. Remote Sens..

[23] Lee J.S. (1980). Digital Image Enhancement and Noise Filtering by Use of Local Statistics. IEEE Trans. Pattern Anal. Mach. Intell..

[24] Lopes A., Nezry E., Touzi R., Laur H. Maximum a Posteriori Filtering and First Order Texture Models in SAR Images. Proceedings of the 10th Annual International Symposium on Geoscience and Remote Sensing.

[25] Yu Y., Acton S. (2002). Speckle Reducing Anisotropic Diffusion. IEEE Trans. Image Process..

[26] Coupe P., Hellier P., Kervrann C., Barillot C. (2009). NonLocal Means-based Speckle Filtering for Ultrasound Images. IEEE Trans. Image Process..

[27] Gonzalez R., Woods R. (2006). Digital Image Processing.

[28] Aharon M., Elad M., Bruckstein A. (2006). K-SVD: An Algorithm for Designing Overcomplete Dictionaries for Sparse Representation. IEEE Trans. Signal Process..

[29] Rubinstein R., Zibulevsky M., Elad M. (2008). Efficient Implementation of the K-SVD Algorithm Using Batch Orthogonal Matching Pursuit.

[30] Horn B., Schunck B. (1980). Determining Optical Flow. Artif. Intell..

[31] Gennert M., Negahdaripour S. (1987). Relaxing the Brightness Constancy Assumption in Computing Optical Flow.

[32] Kanberoglu B., Frakes D. Extraction of Advanced Geospatial Intelligence (AGI) from Commercial Synthetic Aperture Radar Imagery. Proceedings of the Algorithms for Synthetic Aperture Radar Imagery XXIV 2017.

[33] Gao F., Liu X., Dong J., Zhong G., Jian M. (2017). Change Detection in SAR Images Based on Deep Semi-NMF and SVD Networks. Remote Sens..

